# Network-Based Segmentation of Biological Multivariate Time Series

**DOI:** 10.1371/journal.pone.0062974

**Published:** 2013-05-07

**Authors:** Nooshin Omranian, Sebastian Klie, Bernd Mueller-Roeber, Zoran Nikoloski

**Affiliations:** 1 Institute of Biochemistry and Biology, University of Potsdam, Potsdam-Golm, Germany; 2 Systems Biology and Mathematical Modeling Group, Max-Planck Institute of Molecular Plant Physiology, Potsdam-Golm, Germany; 3 Genes and Small Molecule Group, Max-Planck Institute of Molecular Plant Physiology, Potsdam-Golm, Germany; Northwestern University, United States of America

## Abstract

Molecular phenotyping technologies (*e.g.*, transcriptomics, proteomics, and metabolomics) offer the possibility to simultaneously obtain multivariate time series (MTS) data from different levels of information processing and metabolic conversions in biological systems. As a result, MTS data capture the dynamics of biochemical processes and components whose couplings may involve different scales and exhibit temporal changes. Therefore, it is important to develop methods for determining the time segments in MTS data, which may correspond to critical biochemical events reflected in the coupling of the system’s components. Here we provide a novel network-based formalization of the MTS segmentation problem based on temporal dependencies and the covariance structure of the data. We demonstrate that the problem of partitioning MTS data into 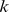 segments to maximize a distance function, operating on polynomially computable network properties, often used in analysis of biological network, can be efficiently solved. To enable biological interpretation, we also propose a breakpoint-penalty (BP-penalty) formulation for determining MTS segmentation which combines a distance function with the number/length of segments. Our empirical analyses of synthetic benchmark data as well as time-resolved transcriptomics data from the metabolic and cell cycles of *Saccharomyces cerevisiae* demonstrate that the proposed method accurately infers the phases in the temporal compartmentalization of biological processes. In addition, through comparison on the same data sets, we show that the results from the proposed formalization of the MTS segmentation problem match biological knowledge and provide more rigorous statistical support in comparison to the contending state-of-the-art methods.

## Introduction

Time-resolved data from different cellular processes hold the promise of identifying the dynamics and relations of key system descriptors mapped into putative metabolic reactions, allosteric regulations, and entire signaling pathways. These data are usually referred to as *multivariate time series* (MTS) since high-throughput technologies allow for simultaneous monitoring of multiple biological entities (*i.e.*, genes, proteins, metabolites) over time. MTS data can capture the dynamics of cellular states constantly adjusting to signals from the environment.

Data from time-resolved experiments contain important temporal and process information, *i.e.*, not only are two (not necessarily consecutive) time points inherently dependent, but also there is a relationship between the instantaneous levels of two measured components due to their proximity in a pathway [1]. An important problem in systems biology is then that of developing methods which would allow for MTS-driven automated construction of time-resolved models that could explain the observed multi-level dependencies [2].

An apparent solution is to determine a representation of the MTS data by which the transient states, corresponding to particular cellular phases, can be extracted. *Time segmentation* is one solution whereby a *single* time series 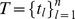 of length 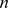 is first partitioned into 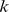 non-overlapping contiguous segments, 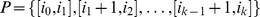, 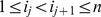, 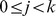, that span the whole series, *i.e.*, 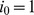 and 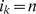. Each segment is represented by either a single quantity, *e.g.*, the mean/median of the time series elements in the segment or the slope of the line yielding the best fit [3]. The difference between a given segment and its representative is measured by using some distance measure 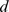 (*e.g.*, Euclidean distance).

The Single 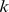-Segmentation problem is that of determining the partition 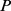 of a given time series into 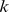 segments together with the corresponding segment representatives 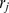, 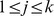, which minimize the following objective function 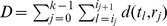. The Single 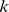-Segmentation problem can be solved in polynomial time in the order of 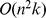 by using dynamic programming [3], [4]. Moreover, for a long time series (*i.e.*, large 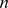), there exist algorithms which solve the problem in subquadratic time with provable constant approximation ratios [5], [6]. The problem has also been formalized and efficiently solved in a Bayesian framework [7], and has found various applications in data mining, classification, and change-point detection (see the review [8]). In contrast, the related Single 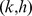-Segmentation problem of selecting 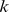 from a set of 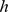 given representatives to optimize the objective 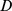 is NP-hard when 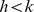, and an 3-approximation algorithm has been proposed in the case when 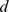 is the Euclidean distance [9].

While in the Single 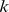-Segmentation problem, the partition of a time series is induced by the chosen distance measure with respect to a well-defined representative, this is often not the case when *multiple* time series are considered. Finding a partition in which multiple time-resolved variables unanimously agree is nontrivial: What constitutes a segment in one variable may not be a segment in another. Moreover, the changes in time-resolved behavior of different variables may not follow the same scale. In addition, to provide a relation to well-established statistical approaches, it is necessary that the segmentation captures the changes in the covariance structure of the MTS data. Therefore, the generalization of the Single 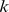-Segmentation problem to MTS data has several potential applications, such as: inferring the critical events occurring in temporally changing systems, detecting periodic or unusual patterns in system’s functions [10], [11], and extracting temporal abstractions for model development [12]–[15].

The existing approaches for segmentation of MTS data are heuristic, and can be classified into three groups based on the employed methods: (1) clustering, (2) graphical models, and (3) genetic algorithms. One of the most important issues with MTS segmentation is the assessment of the resulting partition of segments, as established reference solutions do not exist. All of the existing heuristics thus postulate that an adequate MTS segmentation optimizes a pre-specified function/measure whose solution is in turn regarded as a reference. In a biological setting, the reference state is usually obtained from expert knowledge, one which we rely in this study.

The clustering approaches rely on the homogeneity assumption within segments. They model the segmentation problem of MTS data by grouping time points with the constraint that the data in a cluster must belong to successive time points [11]. Another method based on clustering relies on finding segments for which the distribution of clustered entities approaches the uniform distribution [12], [15]. While this state-of-the-art method has found application in automated model reconstruction, its outcome depends on supervised selection of parameter values. Some of these parameters include the minimum, 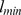, and maximum, 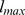, length of segments. Approaches based on 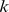-nearest neighbor search in conjunction with common principle components [16], Bayesian clustering [17], fuzzy maximum likelihood clustering of MTS data have also been investigated [18].

A combination of graphical models and maximum likelihood estimation (MLE) has been considered in the second class of methods for MTS segmentation. The idea is to capture mutual dependencies between multiple time series while considering the temporal dependencies within individual series. To this end, the time series are modeled as a special class of random processes [10], [19]–[21]. In the third class, MTS segmentation is addressed with genetic algorithms by which an objective function is optimized. For instance, in [22], Single 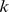-Segmentation is extended to MTS data by considering the slope variance for each segment as an objective function to be minimized. Moreover, the change of the cross-correlation between two variables has also been used as an optimization criterion [23].

Here we propose a network-based formulation for segmentation of MTS data. Our premise is that cellular transition states are reflected in the changes of multiple interrelated biological entities, which can be effectively captured via networks reconstructed from the data. In this way, one accounts for the dependence not only of time points but also of the considered entities. We then investigate to what extent the properties of the reconstructed networks reflect the transition states. Given a polynomially computable distance measure, we demonstrate that finding the partition with minimum number of segments which maximize the sum of distances over all consecutive segments can be solved in polynomial time. This problem can be solved by determining the longest path in a directed acyclic graph derived from the MTS data. In addition, we propose a breakpoint-penalty (BP-penalty) which penalizes the inclusion of breakpoints. Coupling of the BP-penalty with the maximization of a distance function allows the investigation of the interplay between the weight of a path, derived from the MTS data, the number of considered segments as well as the distribution of segment lengths. Further, we explore the advantages and shortcomings of using the proposed formulation in obtaining biologically meaningful interpretations. The resulting framework is shown to outperform the state-of-the-art methods on synthetic as well as transcriptomics MTS data sets from *Saccharomyces cerevisiae* (yeast).

## Methods

### Network Properties

Several network properties of biological networks, obtained from existing biological knowledge or reconstructed from data, have already found important applications in biological studies [24], [25]. Network properties can be defined on global, local, and local-global level depending on the information required for their computation and the network entities to which they pertain. Global network properties, such as: the number of edges, number of nodes, independence number, or chromatic number, characterize and require knowledge of the entire network. On the other hand, the degree of a node 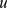 is a local property defined as the number of edges incident on 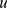. It can be used to quantify the overall activity of the biological entity, modeled by the node 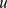, in experiments from which the network has been reconstructed. This network property can be extended on the global level by taking the average degree over all nodes.

Betweenness centrality of a node 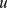 is defined by the number of shortest paths which pass through 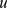 [26]. This is a local-global property as its computation requires information about the entire network, but characterizes a single node. It can be seen as a measure of the control power the node has over information transfer in the network [27]. The average betweenness centrality over all nodes can then be regarded as a global property for the network. Closeness centrality of a node 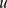 is defined by the inverse of the average length of the shortest paths to all other nodes in the given network [28]. This local-global property can be regarded as a measure of how well the node is integrated in the network. Local and local-global network properties, such as: degree and betweenness centrality, have been used to characterize and predict essential genes in protein-protein interaction and co-expression networks [29], [30]. Moreover, other centrality measures have been associated to genes and proteins playing a key role in cellular processes [27], [31], [32].

### Distance Measures for Network Properties

The distance between two graphs, 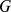 and 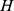, over the same set of nodes, *i.e.*, 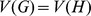, can be expressed in terms of: (1) local(-global) and (2) global network properties. Given a network 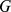, let 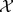 denote a local(-global) property, and let 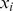 be the value of the property 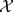 for a node 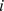. A network 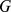 on 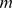 nodes can then be described by the vector 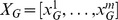. The distance between two networks 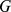 and 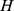, can then be defined with the 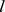-norm of the vector 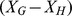:
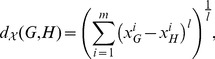
(1)where 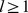. Note that for 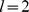, Eq. (1) is the Euclidean distance between the vectors 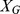 and 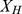. For the second case, let 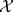 be a global property, and let 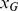 denote its value for a graph 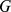. The distance between two networks 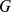 and 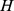 can be defined in terms of the global property as follows:
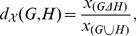
(2)where 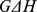 and 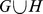 denote the symmetric difference and the union of 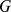 and 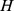, respectively. For instance, if 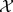 is the relative density of a network 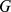, defined as the ratio of the number of edges to the number of nodes in 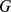, then the distance is:



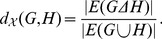
(3)Note that a greater value for the distance measure in Eq. (3) implies a sparser network intersection.

### Network Construction and Problem Definition

Time-resolved high-throughput data are usually summarized in a matrix 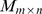, where 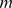 is the number of investigated biological components and 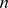 is the number of different measurements at the corresponding time points. Thus, each row of the matrix 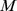 represents a time series data profile for a biological entity. In the simplest case, network representations can be efficiently extracted by applying a similarity measure 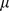 (*e.g.*, Pearson correlation, Euclidean distance, mutual information) on all pairs of data profiles. This yields a similarity matrix 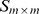. One can then build a network 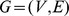 with 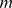 nodes, corresponding to the biological entities; there is an edge between two nodes 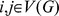 if and only if the element 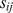 of 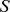 is above an *a priori* estimated threshold 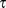.

The threshold 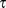 is derived by permutation test such that it ensures a statistical significance at level 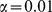 of the observed correlation between the time series profiles of two biological entities. However, a simple randomization strategy neglects the time-course nature of the employed datasets, and, thus, ignores the statistical dependence of successive time points, effectively reducing the degrees of freedom. Therefore, we employ a randomization strategy that retains the dependence of adjacent time points in the permuted data [33]. As a result, the obtained null distributions for each dataset exhibit a heavier tail as compared to distributions based on simple shuffling. As a consequence, this results in a higher value for the threshold 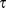 required for significance when keeping dependencies in the original data. In the same fashion, one can reconstruct a similarity matrix 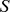 and the resulting graph 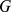 by considering only a subset of consecutive columns corresponding to a segment between two time points.

Given MTS data over 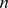 time points, there are 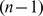 possible segmentation positions. For a fixed segmentation position 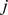, 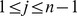, the possible number of segments to the left and to the right of this position are 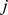 and 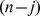, respectively. The segments to the left and right of position 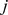 can be combined in 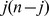 pairs. Each pair of segments allows for the reconstruction of two networks 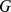 and 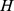, from which values for the distance measures in Eqs. (1) and (2) can readily be computed.

Let 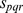 denote the value for the distance measure for graphs 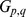 and 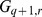 reconstructed from the MTS data on the segments 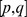 and 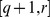, 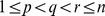, respectively. Note that one has to calculate the distance measure for each of the 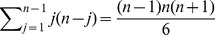 segment pairs. We now define the following bi-optimization problem:

Multiple Segmentation (MULTSEG).

INSTANCE: Given an integer 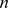 and 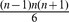 real positive weights 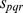.

OBJECTIVES: Determine the ***minimum*** number 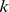 of weights 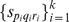, 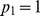, 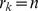, 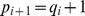 and 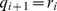, where 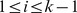, which ***maximize*** 
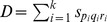
.

**Figure 1:**
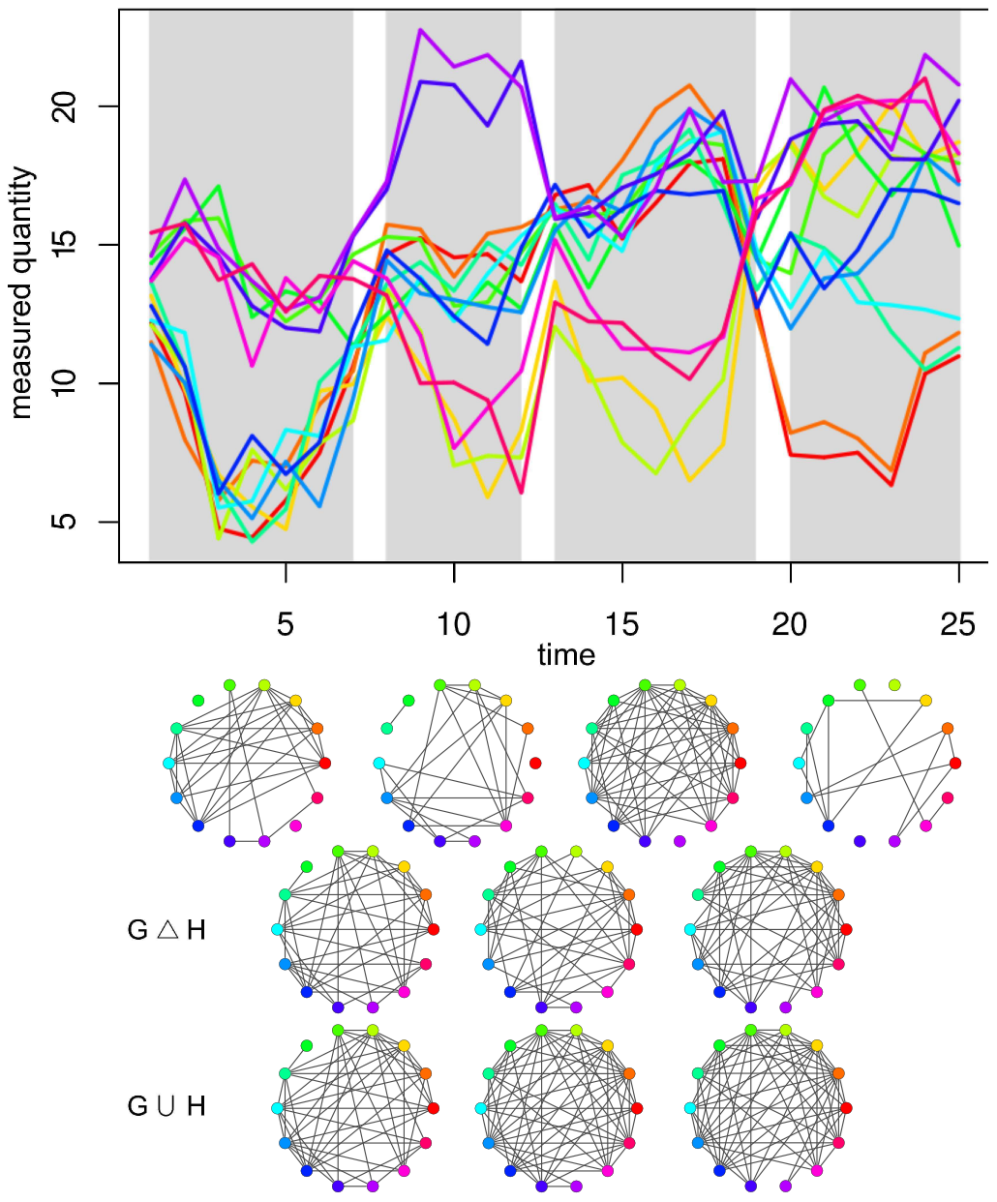
Illustration of the MULTSEG problem. (Upper panel) 14 time series over 25 time points; (Middle panel) Networks reconstructed from the shown series. The networks correspond to the 4 optimal time series segments, depicted with light grey rectangles in the upper panel. The color coding of nodes correspond to the colors of the time series; (Lower panel, the last two rows) Symmetric difference and union networks from the consecutive segments resulting in the optimal value of 2.40 for the objective 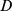, with relative density as a distance measure.

As an illustration, we consider 14 time series over 25 time points shown in the upper panel of Fig. 1. There are 2600 pairs of segments to consider, for which 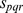 can be determined in terms of network properties according to Eqs. (1) and (2). If 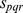 is obtained with the relative density as a global network property, according to Eq. (3), the solution to the MULTSEG is 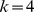 segments resulting in the maximum value 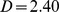. In this paradigmatic example, the networks are shown below each time series segment, colored grey in Fig. 1. The symmetric difference and union of networks for all pairs of consecutive segments used in obtaining the value of 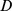 are visualized in the last two rows of Fig. 1, denoted by 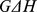 and 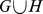, respectively.

### Polynomial Algorithm for MULTSEG

**Figure 2:**
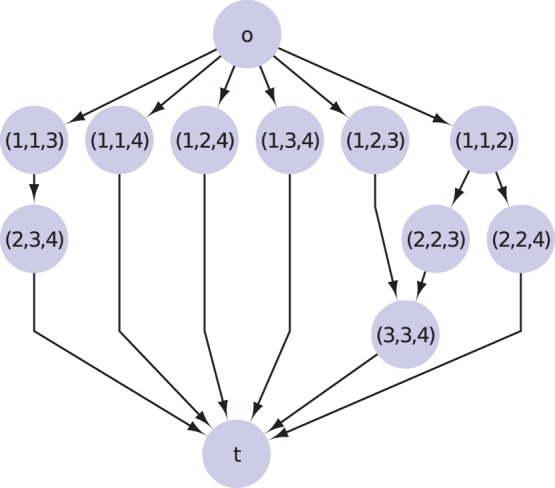
Directed acyclic graph (DAG) used as input in Algorithm 1 (Fig. 3). The DAG 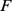 for 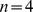 time points is depicted. It contains 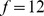 nodes, including the special nodes 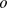 and 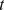. The label 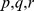 of each node corresponds to the time points 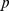, 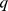, and 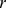.

In the following, we show that the MULTSEG problem is polynomially solvable for distance measures which are computable in polynomial time. To this end, we first transform an instance of the problem into an edge-weighted directed acyclic graph (DAG), 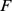, as follows: (1) include two special nodes, a source 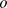 and a target 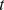, (2) for each of the 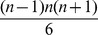 values 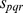, establish a corresponding node 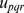, (3) there is a directed zero-weight edge from 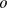 to each 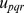, where p = 1, (4) a directed edge from 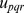 to 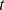 is included if 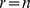, and is assigned a weight of 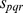, and, finally, (5) a directed edge of weight 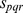 is established from node 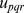 to node 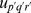 if and only if 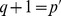 and 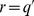. An illustration of the resulting graph for 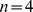 is given in Fig. 2. The resulting directed acyclic graph 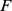 has 
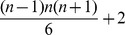
 nodes and 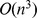 edges. Finding the minimum number of weights 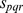 as the first objective while maximizing the second objective 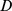 is then equivalent to determining the path of maximum weight with the smallest length (*i.e.*, the minimum number of edges) in 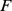.

**Theorem 1:** The MULTSEG problem can be solved in polynomial time in the order of 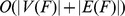.

**Figure 3:**
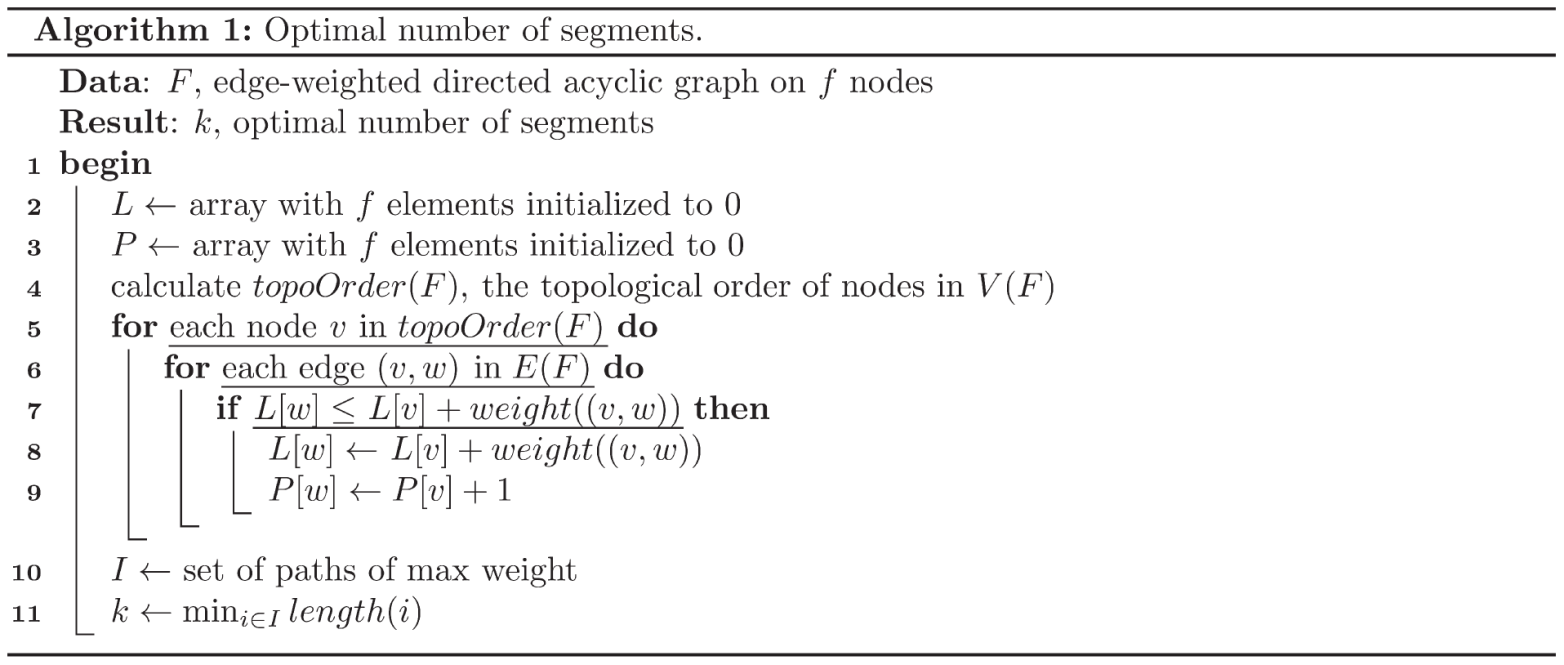
Algorithm 1 - Optimal number of segments. It presents the algorithm for computing the optimal segmentation based on the longest path in a directed acyclic graph.

Proof. Let 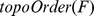 denote the sequence of nodes of 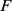 in topological order. A topological ordering of a directed acyclic graph is a linear ordering of its nodes in which each node appears before all other nodes to which it has outgoing edges. It can be obtained in polynomial time in the order of 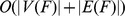 [34]. Let 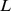 denote the array with 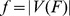 elements initialized to zero, and 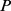 be a predecessor array. Furthermore, let the weight of a path in 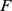 be the sum of edge-weights on the path and the length of the path be the number of edges. The largest weight of a path from the source 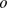 to the target 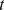 equals the sum of the largest weight of the path from 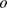 to a predecessor of 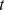 and the weight of the edge from the predecessor to 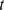. Determining the path of maximum weight in 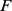 can be solved by the dynamic programming approach given in Algorithm 1 (Fig. 3). The number of segmentation positions 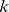 can then be determined as the minimum number of edges on the path of maximum weight in 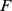. The claim follows from the fact that the algorithm considers all nodes and edges.

The algorithm’s performance can be improved if one requires that the segment length be greater than a given threshold. This strategy can be used to ensure that the network representatives for the considered segments are obtained in statistically rigorous manner. While determining the path of largest weight with the smallest number of segmentation points provides a theoretically optimal way of finding a network-based segmentation of MTS data, it may not carry biologically relevant information (see Supporting Information S1 for examples). This is due to the fact that the DAG formulation does not consider the coupling between the weight 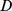 and the length of a path. Therefore, such an approach may result in paths of maximum weight which are much longer compared to the segmentations slightly away from the global optimum. To resolve this issue, we provide a (biological) penalization for paths in the considered DAG.

### Formulation of the Problem with Penalty

In the solution to the MULTSEG problem given in Algorithm 1 (Fig. 3), the edge between the node 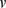 and its successor node 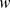, in the constructed DAG, will be considered for inclusion to the optimal path (*i.e.*, path of maximum weight) if it fulfills the inequality in Line 7. Therefore, the optimal path from source 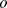 to node 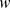 is calculated by the following:
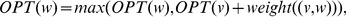
(4)where 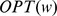 and 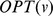 denote the optimal sum of weights of the paths from source 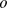 to the node 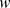 and 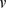, respectively. Note that in Eq. (4), the weights are added irrespective of the number of segments in the optimal path. Therefore, this formulation does not consider simultaneous optimization for the number of breakpoints (*i.e.*, directed edges from the DAG).

To address this problem while still maintaining the generality of the network-based dynamic programming formulation of the MTS segmentation, we define the penalized version of the optimal path (*i.e.*, path of maximum weight) algorithm Algorithm 1 (Fig. 3) which considers a BP-penalty for adding a new segment (breakpoint) to the path.

There are two criteria which can be considered in the formulation of the BP-penalty: (1) the number of segments or (2) the distribution of lengths of the segments included in a path. In general, the expression for the optimal path from source 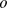 to node 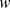 is modified to Eq. (5), whereby the optimal path from 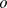 to 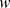 is penalized for inclusion of the breakpoint 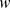, as follows:
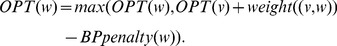
(5)


Lines 7 and 8 in Algorithm 1 (Fig. 3) are accordingly modified. If criterion (1) is used, the BP-penalty for adding the node 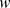 to the optimal path here will be calculated based on the following:
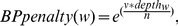
(6)where 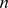 is the maximum possible number of breakpoints for the given time series, 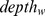 is the number of segments in the path from source 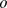 to the node 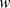 (*i.e.*, it corresponds to the depth (level) of the node 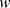 in the DAG) and 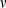 is a tuning parameter which alters in the predefined range. The lower and upper bounds for the tuning parameter 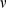 are assigned based on the weight of the path (*i.e.*, distances between pairs of the segments), such that the lower bound is equal with 
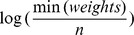
 and the upper bound is, 
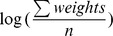
. We note that while the penalty function may assume other forms, it should relate to the value used in defining the weight of a path. This requirement stems from the observation that there is no trivial reference which the weight of a path should satisfy, unlike in other problems readily solvable by dynamic programming (*e.g.*, segmenting least squares [4]).

The penalty of a path 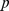 is the sum of BP-penalties for 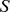 segments and based on Eq. (6) is then equal to:
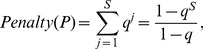
(7)where 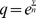.

If criterion (2) is used, the BP-penalty for adding the node 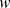 to the optimal path here is defined as:
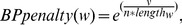
(8)where 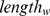 is the number of time points in the new segment needed for including the node 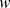 in the path. The lower and upper bounds of the tuning parameter 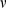 are estimated the same as for the first criterion. The penalty of a path 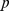 is the sum of BP-penalties for 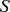 segments:
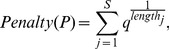
(9)where 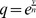, 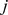 is the number of segments (breakpoints) in the path. Like with the first criterion, here, too, the BP-penalty function may assume another form, depending on applications, but should nevertheless consider dependence on the range of the values for the employed network property.

In the formulation of the first criterion, the penalty grows with the number of segments. On the other hand, by the formulation of the second criterion, the penalty decreases with the length of the segment (due to the reciprocal relationship in Eq. (8)).

Our goal is to find a path corresponding to MTS segmentation which then optimizes the function that combines the weight of the path with the number of segments or the distribution of segment lengths. Our tests with synthetic and real-world data indicate that the first criterion for penalizing a path may be better suited to address the penalized version of the MTS segmentation problem (see Supporting Information S1). Therefore, the results presented and discussed in the remaining sections are based on BP-penalty according to Eq. (6), above.

## Results

### Synthetic Data

**Figure 4:**
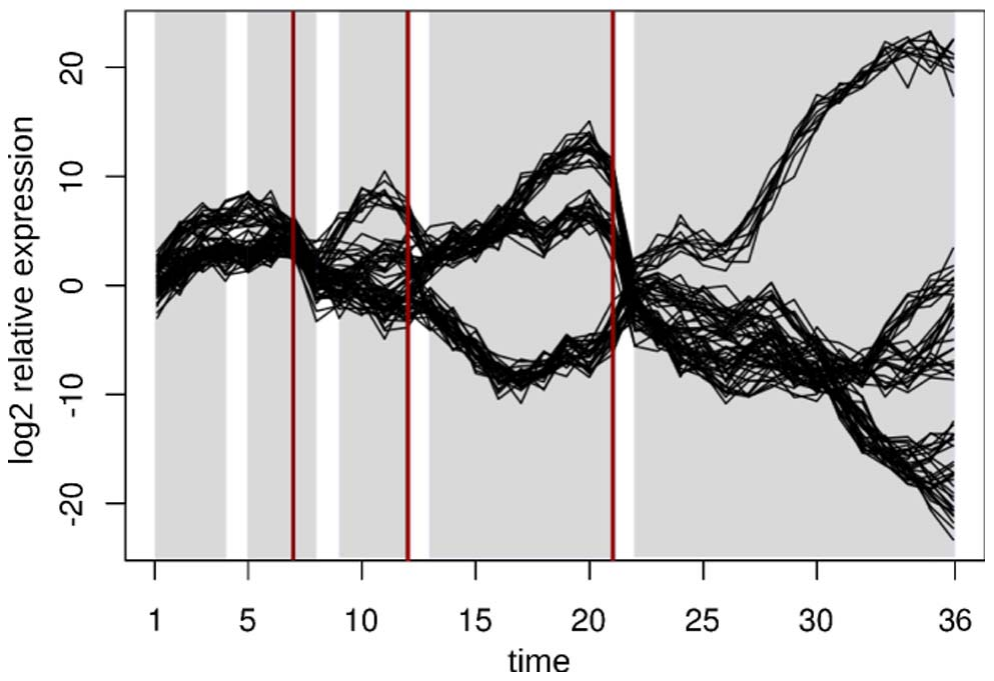
Illustration of the segmentation for synthetic data with relative density as network property. The resulting partitions are highlighted in light grey and the simulated segmentation points are marked with red bars.

To investigate the performance of the algorithm, we created synthetic time series data for 70 variables over 36 time points (see Fig. 4). The segmentation points correspond to the time points 7, 12 and 21. To create these segmentation points, a number of data profiles were generated for each segment by simulating a zero-mean autoregressive moving average (ARIMA) model by using arima.sim in R [35]. The number of profiles simulated for the four segments, [1], [7], [8], [12], [13], [21], [22], [36], was set to 2, 6, 3, and 7, respectively. Each of the 70 variables was obtained by randomly sampling a characteristic data profile in each segment. In addition, a normally distributed error term, 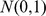, was added to the sampled profile value at each time point. Finally, to simulate the temporal dependence between two adjacent segments, the boundaries between two segments of each variable were smoothed using a discrete linear filter approximating a Gaussian kernel. To this end, for each obtained profile, the simulated measurement 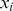 at time-point 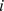, where 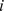 is the left boundary of each segment, *i.e.*, 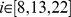, was replaced by 
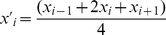
.

The proposed theoretical framework was used in the analysis of the synthetic data with four different network properties, namely: degree (local), closeness and betweenness (local-global), and relative density (global). The results are presented in Table 1, and the best performing result is graphically depicted in Fig. 4, for 20 equidistant values for the tuning parameter in the intervals given in Table 1. The predictions from the relative density, as a global network property, are closest to the simulated segmentation. As expected, the global properties provide more meaningful results since they capture the global changes in the network topology over the considered time domain. Although, the predictions of the simulated segmentation included an additional time point, this can be explained by the structure of the DAG. By the proposed penalization of paths, the BP-penalty is calculated for adding a new breakpoint to the optimal path, while in traversing the DAG from the source node 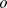 to its first neighbors, two new breakpoints are necessarily added. Thus, the BP-penalty for adding this node to the path is not taken into account. Therefore, it is expected that the first actual segment in the time series is split into two finer segments. In contrast, the best solution from the state-of-the-art method of [15] was obtained by setting the parameters 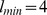 and 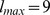; however, this solution is also included the extra breakpoint at the time point 29, which shows that both algorithms perform almost the same (see Table S1).

**Table 1:** Optimal segmentation for synthetic data.

Network property	Type	*k*	Segments	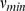	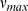
relative density	G	5	[1]–[4],[5]–[8],[9]–[12],[13]–[21],[22]–[36]	0.05	6.00
degree	L	5	[1]–[4], [5]–[8], [9]–[12], [13], [24], [25]–[36]	1.50	11.13
closeness	LG	4	[1]–[4], [5]–[15], [16]–[20], [21]–[36]	0.05	4.47
betweenness	LG	7	[1]–[4], [5]–[8], [9]–[12], [13]–[17], [18]–[21], [22]–[29], [30]–[36]	1.06	12.10
**Existing method**		***k***	**Segments**	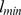	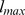
Ramakrishnan *et al.* [15]		5	[1]–[7], [8]–[12], [13]–[21], [22]–[29], [30]–[36]	4	9

The upper part of the table shows the result of the optimal segmentation for synthetic data based on dynamic programming, while the lower part contains the result based on the method of Ramakrishnan *et al.* [15]. In the upper table, the first and second columns show the name and the type of network properties used to determine the distances: G stands for global, L for local, and LG for local-global. The third column includes the number 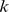 of segments that maximize the objective 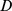 with the dynamic programming approach. The resulting segments are given in the forth column, while the fifth and sixth columns contain the corresponding values of lower (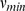) and upper (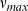) bound of the tuning parameter 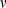. The lower part also includes minimum and maximum length of the segments, i.e., 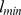 and 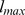, as parameters of the contending method.

### Yeast’s Metabolic and Cell Cycles

Motivated by the accurate predictions from applying the framework on the synthetic data set, we next investigated the MTS segmentation of transcriptomics data sets from the *Saccharomyces cerevisiae* metabolic cycle [36] (YMC), cell cycle [37] (YCC), and the experiment capturing the effect of oxidative stress, induced by hydrogen peroxide (HP), on the yeast’s cell cycle [38]. In all data sets, we filtered the genes which: (1) contain missing values, (2) have not been annotated with any GO term, and (3) have coefficients of variation smaller than 1.

We first investigate the transcriptomics time series from YMC data set. The yeast metabolic cycle consists of the following three successive phases spanning each 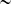 5 h: (1) a reductive charging (R/C) phase, involving non-respiratory metabolism (glycolysis and fatty acid oxidation) and protein degradation, (2) oxidative metabolism (OX), in which respiratory processes are used to generate adenosine triphosphate (ATP), (3) reductive metabolism (R/B), marked by a decrease in oxygen uptake and dominance of DNA replication, mitochondrial biogenesis, ribosome biogenesis, and cell division [36]. The data set includes the time-resolved expression of 6555 genes (with 9335 probes) over 36 time points (separated by 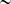 25-min intervals) over three consecutive metabolic cycles. Clustering of the obtained transcript profiles was employed in Tu *et al.* [36] to show that YMC controls the timing of key cellular and metabolic processes to allow coordination of anabolic and catabolic processes for efficient energy production and usage. Therefore, this data set can serve as a benchmark for testing of our proposed algorithms for MTS segmentation.

With the filtering step, the number of genes was reduced from 6555 to 255. The latter were employed to determine the segmentation based on four network properties: degree, betweenness, and closeness, according to Eq. (1) with 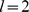, as well as the relative density, given in Eq. (3). Only segments of length at least 4 were considered in order to ensure statistical significance of the Pearson correlation used in network reconstruction. We estimated the thresholds for the Pearson correlation over all considered segment lengths, at significance level 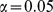, by employing an empirical permutation test and the randomization procedure from Kruglyak and Tang [33], which allows us to consider a dependence structure of adjacent time points.

**Figure 5:**
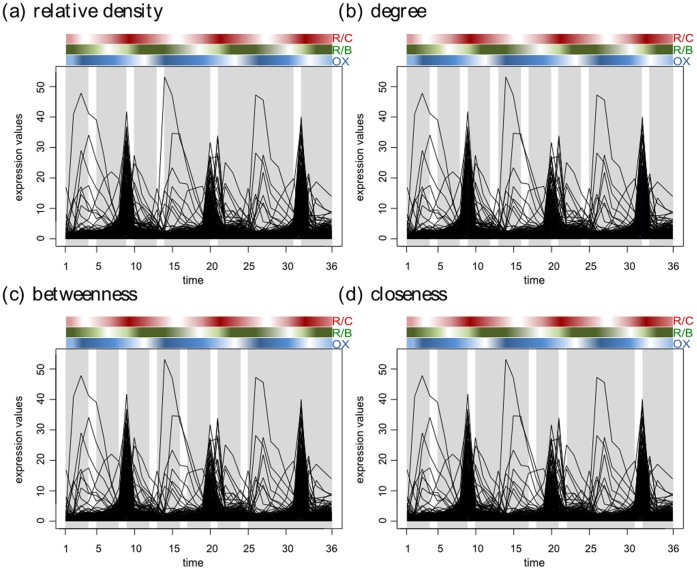
Segmentation for yeast’s metabolic cycle. The partitions found by applying our method are highlighted in light grey. The phases of the yeast metabolic cycle are indicated with colored rectangles above each panel following Tu *et al.* [36]. R/C stands for reductive charging, OX oxidative metabolism, and R/B, reductive metabolism. (a) shows the segmentations caught by relative density as global property; (b) illustrates the segmentations based on degree; (c) and (d) demonstrate segmentations with local-global properties, betweenness and closeness, respectively. The segmentations in panel (a) performs particularly well due to the global changes in the form of global cycles in the data set from yeast.

The range for the tuning parameter for each used network property together with the resulting segmentations and number of segments are summarized in Table 2. Due to the presence of recurrent changes on the global level, two segmentation points, corresponding to time points 12–13 and 24–25 and delineating the three considered cell cycles, should be detected. In addition, due to the presence of the alternation phases in the metabolic cycle, each of the three cycles should contain at least one more segmentation point. Altogether, this biological reasoning implies the existence of six to seven segmentation points in the investigated time domain. Inspection of the results in Table 2 indicates that when using the BP-penalty, the degree resulted in the most biologically meaningful prediction for the segmentation points in the first two cell cycles, where the starting of each of the three phases is nicely delineated. A similar behavior is observed for the betweenness centrality. However, none of the properties results in the identification of an additional breakpoint in the third cycle. The method of Ramakrishnan *et al.* [15] with 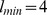 and 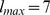 (Table S2 and Figure S1) also results in eight segments which resemble our results (Fig. 5) particularly for the first two cell cycles.

**Table 2:** Optimal segmentation for data from yeast.

Network property	Type	*k*	Segments	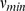	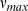
relative density	G	6	[1]–[4],[5]–[9],[10]–[13],[14]–[20],[21]–[31],[32]–[36]	0.05	5.60
degree	L	8	[1]–[4],[5]–[8],[9]–[12],[13]–[16],[17]–[20],[21]–[24],[25]–[32],[33]–[36]	4.35	13.50
closeness	LG	6	[1]–[4],[5]–[9],[10]–[17],[18]–[21],[22]–[31],[32]–[36]	0.05	4.24
betweenness	LG	6	[1]–[4],[5]–[8],[9]–[12],[13]–[20],[21]–[24],[25]–[36]	3.80	14.52
**Existing method**		***k***	**Segments**	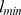	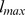
Ramakrishnan *et al.* [15]		8	[1]–[6],[7]–[10],[11]–[14],[15]–[18],[19]–[22],[23]–[26],[27]–[31],[32]–[36]	4	7

The upper part of the table shows the result of the optimal segmentation for synthetic data based on dynamic programming, while the lower part contains the result based on the method of Ramakrishnan *et al.* [15]. In the upper table, the first and second columns show the name and the type of network properties used to determine the distances: G stands for global, L for local, and LG for local-global. The third column includes the number 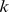 of segments that maximize the objective 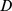 with the dynamic programming approach. The resulting segments are given in the forth column, while the fifth and sixth columns contain the corresponding values of lower (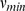) and upper (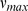) bound of the tuning parameter 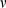. The lower part also includes minimum and maximum length of the segments, i.e., 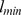 and 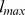, as parameters of the contending method.

We next analyzed the results for the other two data sets, YCC and HP. As summarized in the Tables S3 (for YCC) and S4 (for HP), our method could identify coarser segments typical for the two investigated processes. In contrast, the contending method results in much finer partitions, in which the segments often contain only three time points, for which statistical significance of the findings is difficult to establish. Each yeast cell cycle (YCC) includes the following phases: M/G1, G1, S, G2, and M, such that the M/G1, G1 and S phases last 2 time points each while the G2 phase lasts only one time point, as described in Ramakrishnan *et al.* [15]. This corresponds to the following segmentation for the YCC data set: [1]–[3],[4]–[7],[8]–[11],[12]–[14],[15]–[18]. As shown in Table S3, the segmentation based on the relative density and betweenness matches the expected M/G1, G1, and S phases, but lumps G2 and M together due to the lower bound on the segment length (of 4). In contrast, the contending method provides a uniform distribution of segment lengths, which coincides to the parameter 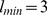, employed in this setting. Moreover, similar to the YMC data set, the segmentation from betweenness in the case of the oxidative stress induced by hydrogen peroxide (HP) supports the biological evidence for four phases of the cell cycle, namely, G1, S, G2, and G2/M (see Table S4).

## Discussion

Biological systems are exposed to perpetual changes of environmental conditions to which they adapt via complex mechanisms. Analysis of MTS data can be used to identify the key biological processes involved in the adjustment of the cellular states. Thus, segmentation of time series lends itself as a means for automatic discovery of the transition states leading to cell vitality.

Here we provided a network-based formalization of the MTS segmentation problem following the dynamic programming approach, where we investigated the differences of network properties upon segmentation, and examined the extent to which transient cellular states are reflected in the chosen network representation. The framework relies on distance measures based on local, local-global, and global network properties. We presented polynomial-time algorithms for the problem of determining the segments which maximize the objective function–the sum of the distances between networks reconstructed from consecutive time segments. In addition, we proposed path penalization to simultaneously consider the number of segments as a factor in determining the optimal path for segmentation. Moreover, we demonstrated that the penalized version of the longest path algorithm allows extraction of biologically meaningful paths on real data sets, as judged by expert knowledge. The predictions from the empirical analysis of synthetic data, specifically tailored for MTS segmentation, and transcriptomics data from yeast showed that local-global network properties can be used to distinguish changes dominate the alteration of the system. Our analysis highlights that even simple distance measures based on relative network density can fairly accurately determine the first two phases of the yeast metabolic cycle.

In addition, as shown in the Supporting Information S1, we demonstrated that the proposed method reveals the phases based on a data sets from yeast’s cell cycle experiment and the phases of the cell cycle in oxidative stress induced by hydrogen peroxide, largely supported by local-global properties, like in the case of the yeast metabolic cycle. Therefore, these findings further demonstrate that the change of network properties over time caries important biological information with respect to segmentation of MTS data. Furthermore, it remains to investigate the results from applying the proposed method to other high-throughput data and their combinations.

The novel formulation of the problem requires that the distances between networks reconstructed from each pair of consecutive segments are known *a priori*. This is of practical importance since the networks can be pre-computed and stored for further analysis. The dynamic programming formulation can be easily obtained, since our solutions require building an edge-weighted directed acyclic graph. We believe that our approach for using network-based segmentation is a first necessary step towards determination of patterns in the dynamics of biological processes from temporal data sets, which will lead to automated model extraction.

## Supporting Information

Figure S1
**Segmentation for yeast’s metabolic cycle based on the method of Ramakrishnan** ***et al.*** [**15**] **.** The partitions found by applying the method of Ramakrishnan *et al.* [15] are highlighted in light grey. The phases of the yeast’s metabolic cycle are indicated with colored rectangles above each panel following Tu *et al.* [36]. R/C stands for reductive charging, OX oxidative metabolism, and R/B, reductive metabolism. The minimum length 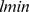 and the maximum, 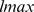 are included in the top left corner.(TIFF)

Table S1
**Optimal segmentation for synthetic data.** The first part of the table comprises the result of the optimal segmentation for synthetic data based on general longest path algorithm Algorithm 1 (Fig. 3). The second and the third parts show the results based on penalized longest path algorithm using number of segments and distribution of length of the segments to calculate the penalty of a path, respectively. The lower part contains the result based on the method of Ramakrishnan *et al.* [15]. In the first part of the table, the first and second columns show the name and the type of network properties used to determine the distances: G stands for global, L for local, and LG for local-global. The third column includes the number 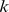 for each of the three methods and the resulting segments are given in the forth column. The fifth and sixth columns in the second and third parts present the values of lower (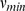) and upper (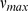) bound of the tuning parameter 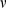 with dynamic programming approach. The lower part also includes minimum and maximum length of the segments, i.e., 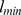 and 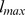, as parameters of the contending method.(PDF)

Table S2
**Optimal segmentation for yeast’s metabolic cycle (YMC) data with the same preprocessing has been applied in Ramakrishnan** ***et al.*** [**15**] **.** The first part of the table comprises the result of the optimal segmentation for synthetic data based on general longest path algorithm Algorithm 1 (Fig. 3). The second and the third parts show the results based on penalized longest path algorithm using number of segments and distribution of length of the segments to calculate the penalty of a path, respectively. The lower part contains the result based on the method of Ramakrishnan *et al.* [15]. In the first part of the table, the first and second columns show the name and the type of network properties used to determine the distances: G stands for global, L for local, and LG for local-global. The third column includes the number 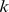 for each of the three methods and the resulting segments are given in the forth column. The fifth and sixth columns in the second and third parts present the values of lower (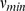) and upper (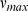) bound of the tuning parameter 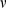 with dynamic programming approach. The lower part also includes minimum and maximum length of the segments, i.e., 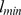 and 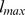, as parameters of the contending method.(PDF)

Table S3
**Optimal segmentation for yeast’s cell cycle (YCC) data.** The first part of the table comprises the result of the optimal segmentation for synthetic data based on general longest path algorithm Algorithm 1 (Fig. 3). The second and the third parts show the results based on penalized longest path algorithm using number of segments and distribution of length of the segments to calculate the penalty of a path, respectively. The lower part contains the result based on the method of Ramakrishnan *et al.* [15]. In the first part of the table, the first and second columns show the name and the type of network properties used to determine the distances: G stands for global, L for local, and LG for local-global. The third column includes the number 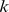 for each of the three methods and the resulting segments are given in the forth column. The fifth and sixth columns in the second and third parts present the values of lower (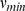) and upper (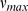) bound of the tuning parameter 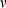 with dynamic programming approach. The lower part also includes minimum and maximum length of the segments, i.e., 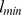 and 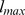, as parameters of the contending method.(PDF)

Table S4
**Optimal segmentation for data from oxidative stress, induced by hydrogen peroxide (HP), on yeast’s cell cycle.** The first part of the table comprises the result of the optimal segmentation for synthetic data based on general longest path algorithm Algorithm 1 (Fig. 3). The second and the third parts show the results based on penalized longest path algorithm using number of segments and distribution of length of the segments to calculate the penalty of a path, respectively. The lower part contains the result based on the method of Ramakrishnan *et al.* [15]. In the first part of the table, the first and second columns show the name and the type of network properties used to determine the distances: G stands for global, L for local, and LG for local-global. The third column includes the number 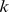 for each of the three methods and the resulting segments are given in the forth column. The fifth and sixth columns in the second and third parts present the values of lower (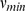) and upper (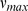) bound of the tuning parameter 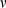 with dynamic programming approach. The lower part also includes minimum and maximum length of the segments, i.e., 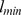 and 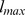, as parameters of the contending method.(PDF)

Supporting Information S1 **It includes a detailed description of the data sets used in the computational analysis.**
(PDF)

## References

[1] Steuer R, Kurths J, Fiehn O, Weckwerth W (2003) Interpreting correlations in metabolomic networks. Biochemical Society Transactions 31: 1476–1478. 14641093 10.1042/bst0311476

[2] Fisher J, Henzinger TA (2007) Executable cell biology. Nature Biotechnology 25: 1239–1249. 10.1038/nbt1356 17989686

[3] Bellman R, Roth R (1969) Curve fitting by segmented straight lines. Journal of the American Statistical Society 64: 1079–1084.

[4] Kleinberg J, Tardos E (2005) Algorithm Design, Boston, MA, USA: Addison-Wesley Longman Publishing Co., Inc., chapter 6.

[5] Guha S, Koudas N, Shim K (2001) Data-streams and histograms. In: Proceedings of the thirty- third annual ACM symposium on Theory of computing. New York, NY, USA: ACM, STOC ’01, 471–475. doi:10.1145/380752.380841. Available: http://doi.acm.org/10.1145/380752.380841.

[6] Terzi E, Tsaparas P (2006) Efficient algorithms for sequence segmentation. In: SDM.

[7] Fearnhead P (2006) Exact and efficient Bayesian inference for multiple changepoint problems. Statistics and Computing 16: 203–213.

[8] Keogh E, Chu S, Hart D, Pazzani M (2003) Segmenting time series: A survey and novel approach. Work 57: 1–21.

[9] Gionis A, Mannila H (2003) Finding recurrent sources in sequences. Proceedings of the Seventh Annual International Conference on Computational Molecular Biology RECOMB 03 123–130.

[10] Yin J, Shen D, Yang Q, Li ZN (2005) Activity recognition through goal-based segmentation. In: Proceedings of the 20th national conference on Artificial intelligence - Volume 1. AAAI Press, AAAI’05, 28–33. Available: http://dl.acm.org/citation.cfm?id=1619332.1619339.

[11] Duchene F, Garbay C, Rialle V (2007) Learning recurrent behaviors from heterogeneous multivariate time-series. Artificial Intelligence in Medicine 39: 25–47. 16935482 10.1016/j.artmed.2006.07.004

[12] Tadepalli S, Ramakrishnan N, Mishra B, Watson LT, Helm RF (2008) Deriving kripke structures from time series segmentation results. In: Discrete Event Systems, 2008. WODES 2008. 9th International Workshop on. IEEE, 406–411. Available: http://dx.doi.org/10.1109/WODES.2008.4605980.

[13] Batal I, Sacchi L, Bellazzi R, Hauskrecht M (2009) Multivariate time series classification with temporal abstractions. Florida Artificial Intelligence Research Society Conference : 344–349.

[14] Picard F, Lebarbier E, Budinska E, Robin S (2011) Joint segmentation of multivariate gaussian processes using mixed linear models. Computational Statistics & Data Analysis 55: 1160–1170.

[15] Ramakrishnan N, Tadepalli S, Watson LT, Helm RF, Antoniotti M, et al (2010) Reverse engineering dynamic temporal models of biological processes and their relationships. Proceedings of the National Academy of Sciences of the United States of America 107: 12511–12516. 20571120 10.1073/pnas.1006283107 PMC2906599

[16] Yang K, Shahabi C (2007) An efficient k nearest neighbor search for multivariate time series. Information and Computation 205: 65–98.

[17] Ramoni M, Sebastiani P, Cohen P (2002) Bayesian clustering by dynamics. Machine Learning 47: 91–121.

[18] Abonyi J, Feil B, Nemeth S, Arva P (2005) Modified gath–geva clustering for fuzzy segmentation of multivariate time-series. Fuzzy Sets and Systems 149: 39–56.

[19] Xuan X, Murphy K (2007) Modeling changing dependency structure in multivariate time series. In: Proceedings of the 24th international conference on Machine learning. New York, NY, USA: ACM, ICML ’07, 1055–1062. doi:10.1145/1273496.1273629. Available: http://doi.acm.org/10.1145/1273496.1273629.

[20] Dobigeon N, Tourneret JY, Scargle JD (2007) Joint segmentation of multivariate astronomical time series: Bayesian sampling with a hierarchical model. IEEE Transactions on Signal Processing 55: 414–423.

[21] Bai J, Perron P (2003) Computation and analysis of multiple structural change models. Journal of Applied Econometrics 18: 1–22.

[22] Graves D, Pedrycz W (2009) Multivariate segmentation of time series with differential evolution. In: IFSA/EUSFLAT Conf. 1108–1113.

[23] Tucker A, Liu X, Ogden-Swif A (2001) Evolutionary learning of dynamic probabilistic models with large time lags. International Journal of Intelligent Systems 16: 621–645.

[24] Albert R (2005) Scale-free networks in cell biology. Journal of Cell Science 118: 4947–4957. 16254242 10.1242/jcs.02714

[25] Sweetlove LJ, Fell D, Fernie AR (2008) Getting to grips with the plant metabolic network. The Biochemical Journal 409: 27–41. 18062772 10.1042/BJ20071115

[26] Freeman LC (1977) A set of measures of centrality based on betweenness. Sociometry 40: 35–41.

[27] Koschuetzki D, Schreiber F (2008) Centrality analysis methods for biological networks and their application to gene regulatory networks. Gene Regulation and Systems Biology 2: 193–201. 19787083 10.4137/grsb.s702 PMC2733090

[28] Freeman L (1979) Centrality in social networks conceptual clarification. Social Networks 1: 215–239.

[29] Jeong H, Mason SP, Barabasi AL, Oltvai ZN (2001) Lethality and centrality in protein networks. Nature 411: 41–42. 11333967 10.1038/35075138

[30] Hahn MW, Kern AD (2004) Comparative genomics of centrality and essentiality in three eukaryotic protein-interaction networks. Molecular Biology and Evolution 22: 7–10. 10.1093/molbev/msi072 15616139

[31] Joy MP, Brock A, Ingber DE, Huang S (2005) High-betweenness proteins in the yeast protein interaction network. Journal of Biomedicine and Biotechnology : 96–103. 10.1155/JBB.2005.96 PMC1184047 16046814

[32] Manimaran P, Hegde SR, Mande SC (2009) Prediction of conditional gene essentiality through graph theoretical analysis of genome-wide functional linkages. Molecular Biosystems 5: 1936–1942. 19763329 10.1039/B905264j

[33] Kruglyak S, Tang H (2001) A new estimator of significance of correlation in time series data. Journal of Computational Biology 8: 463–470. 11694177 10.1089/106652701753216486

[34] Kahn AB (1962) Topological sorting of large networks. Commun ACM 5: 558–562.

[35] Brockwell P, Davis R (1996) Introduction to time series and forecasting. New York: Springer.

[36] Tu BP, Kudlicki A, Rowicka M, McKnight SL (2005) Logic of the yeast metabolic cycle: temporal compartmentalization of cellular processes. Science 310: 1152–1158. 16254148 10.1126/science.1120499

[37] Spellman PT, Sherlock G, Zhang MQ, Iyer VR, Anders K, et al (1998) Comprehensive identification of cell cycle-regulated genes of the yeast Saccharomyces cerevisiae by microarray hybridization. Molecular Biology of the Cell 9: 3273–3297. 9843569 10.1091/mbc.9.12.3273 PMC25624

[38] Shapira M, Segal E, Botstein D (2004) Disruption of yeast forkhead-associated cell cycle transcription by oxidative stress. Molecular Biology of the Cell 15: 5659–5669. 15371544 10.1091/mbc.E04-04-0340 PMC532044

