# Supplementary figures and images for: Network-Based Segmentation of Biological Multivariate Time Series
Source: PLoS One. 2013 May 7;8(5):e62974. doi: 10.1371/journal.pone.0062974 (PMC3646968; doi:10.1371/journal.pone.0062974)

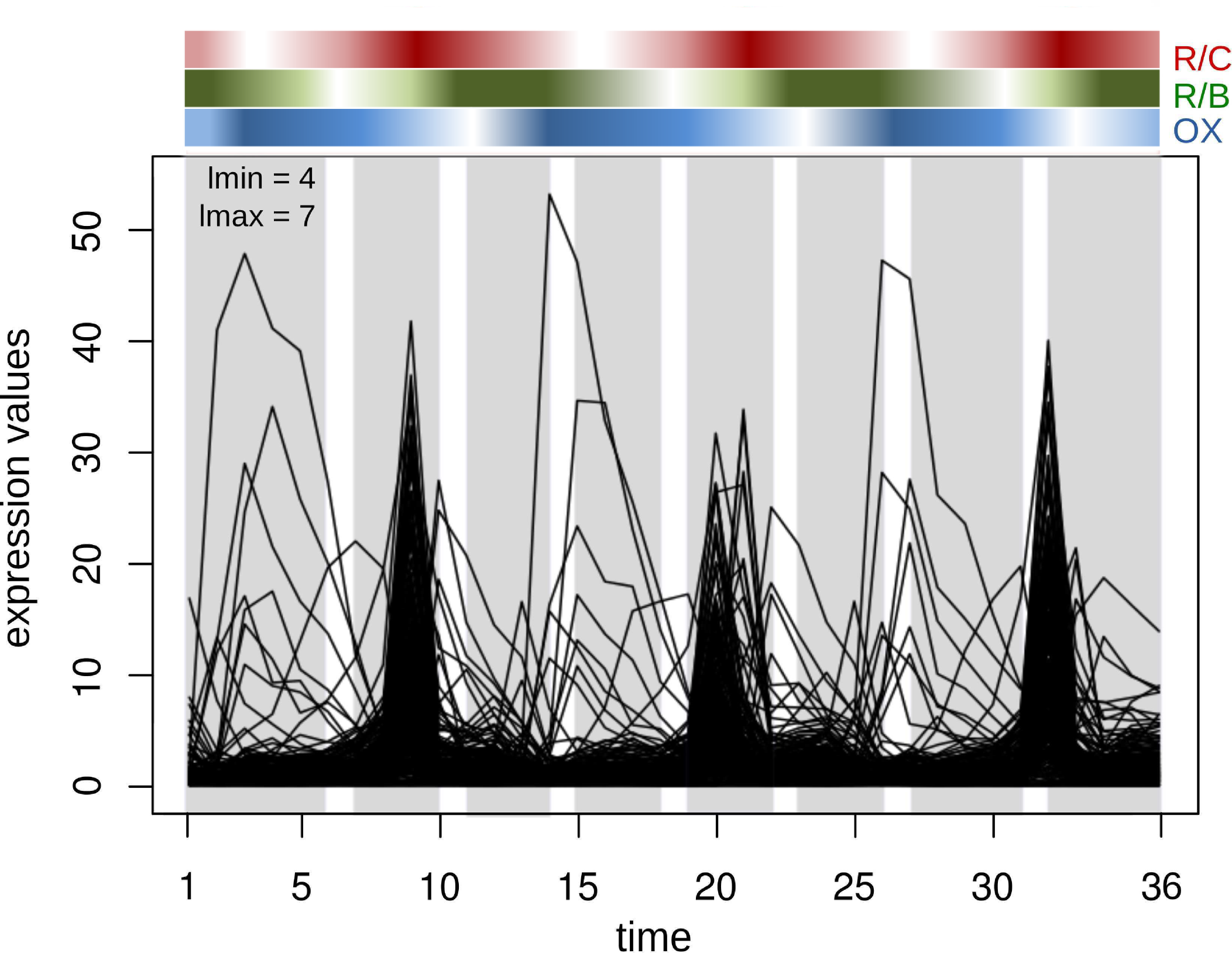

Supplement: Figure S1 — Segmentation for yeast’s metabolic cycle based on the method of Ramakrishnan et al. [15] . The partitions found by applying the method of Ramakrishnan et al. [15] are highlighted in light grey. The phases of the yeast’s metabolic cycle are indicated with colored rectangles above each panel following Tu et al. [36]. R/C stands for reductive charging, OX oxidative metabolism, and R/B, reductive metabolism. The minimum length and the maximum, are included in the top left corner. (TIFF) [file pone.0062974.s001.tiff]
