# Supplementary material for: Network-Based Segmentation of Biological Multivariate Time Series
Source: PLoS One. 2013 May 7;8(5):e62974. doi: 10.1371/journal.pone.0062974 (PMC3646968; doi:10.1371/journal.pone.0062974)
Supplement: Table S3 — Optimal segmentation for yeast’s cell cycle (YCC) data. The first part of the table comprises the result of the optimal segmentation for synthetic data based on general longest path algorithm Algorithm 1 (Fig. 3). The second and the third parts show the results based on penalized longest path algorithm using number of segments and distribution of length of the segments to calculate the penalty of a path, respectively. The lower part contains the result based on the method of Ramakrishnan et al. [15]. In the first part of the table, the first and second columns show the name and the type of network properties used to determine the distances: G stands for global, L for local, and LG for local-global. The third column includes the number for each of the three methods and the resulting segments are given in the forth column. The fifth and sixth columns in the second and third parts present the values of lower () and upper () bound of the tuning parameter with dynamic programming approach. The lower part also includes minimum and maximum length of the segments, i.e., and , as parameters of the contending method. (PDF) [file pone.0062974.s004.pdf]

Table S3. Optimal segmentation for yeast's cell cycle (YCC) data. The first part of the table comprises the result of the optimal segmentation for synthetic data based on general longest path algorithm Algorithm 1 (Fig. 3). The second and the third parts show the results based on penalized longest path algorithm using number of segments and distribution of length of the segments to calculate the penalty of a path, respectively. The lower part contains the result based on the method of Ramakrishnan *et al.* [15]. In the first part of the table, the first and second columns show the name and the type of network properties used to determine the distances: G stands for global, L for local, and LG for local-global. The third column includes the number k for each of the three methods and the resulting segments are given in the forth column. The fifth and sixth columns in the second and third parts present the values of lower (ν_{min}) and upper (ν_{max}) bound of the tuning parameter ν with dynamic programming approach. The lower part also includes minimum and maximum length of the segments, i.e., l_{min} and l_{max}, as parameters of the contending method.

**Algorithm 1 (Fig. 3)**	**Type**	***k***	**Segments**		
relative density	G	4	[1-5],[6,9],[10-13],[14-18]		
degree	L	3	[1-6],[7-14],[15-18]		
closeness	LG	3	[1-4],[5-14],[15-18]		
betweenness	LG	3	[1-5],[6-14],[15-18]		
**Penalized (number)**	**Type**	***k***	**Segments**	ν_{min}	ν_{max}
relative density	G	4	[1-4],[5-8],[9-12],[13-18]	0.05	4.08
degree	L	3	[1-6],[7-14],[15-18]	6.64	12.72
closeness	LG	3	[1-5],[6-14],[15-18]	0.05	5.64
betweenness	LG	4	[1-4],[5-8],[9-14],[15-18]	9.15	15.38
**Ramakrishnan *et al.* [15]**		***k***	**Segments**	l_{min}	l_{max}
		6	[1-3],[4-6],[7-9],[10-12],[13-15],[16-18]	3	5
