# Supplementary material for: Network-Based Segmentation of Biological Multivariate Time Series
Source: PLoS One. 2013 May 7;8(5):e62974. doi: 10.1371/journal.pone.0062974 (PMC3646968; doi:10.1371/journal.pone.0062974)
Supplement: Supporting Information S1 — It includes a detailed description of the data sets used in the computational analysis. (PDF) [file pone.0062974.s006.pdf]

Supporting information S1
Network-based segmentation of biological multivariate time series

Nooshin Omranian[1,2], Sebastian Klie[3], Bernd Mueller-Roeber[1], Zoran Nikoloski[2,*]
1 Institute of Biochemistry and Biology, University of Potsdam, Potsdam-Golm, Germany
2 Systems Biology and Mathematical Modeling Group, Max-Planck Institute of Molecular Plant Physiology, Potsdam-Golm, Germany
3 Genes and Small Molecule Group, Max-Planck Institute of Molecular Plant Physiology, Potsdam-Golm, Germany
∗ E-mail: nikoloski@mpimp-golm.mpg.de

Synthetic data

In this section, we provide additional elaboration of the results from different approaches applied on the synthetic data, described in the main text. The results are succinctly summarized in Table S1, showing the comparison between the obtained segmentations.

Yeast's metabolic cycle

In this section, we provide additional elaboration of the results from different approaches applied on the yeast's metabolic cycle (YMC) data, described in the main text. The results are succinctly summarized in Table S2, showing the comparison between the obtained segmentations.

Yeast's cell cycle

In this section, we provide additional elaboration of the results from different approaches applied on the yeast's cell cycle (YCC) data. With the filtering step, the number of genes was reduced from 6076 to 2071. The latter were employed to determine the segmentation based on four network properties: degree, betweenness, and closeness, and relative density. Only segments of length at least 4 were considered in order to ensure statistical significance of the Pearson correlation used in network reconstruction. We estimated the thresholds for the Pearson correlation over all considered segment lengths, at significance level $\alpha = 0.05$, by employing an empirical permutation test and the randomization procedure from Kruglyak and Tang [33], which allows to consider a dependence structure of adjacent time points.

The characteristics of the resulting segmentations are summarized in Table S3. Based on the work done by Spellman *et al.* [37], with the relative density, which is a global property, we could discern the cycles in the system. Each cycle includes the following phases: M/G1, G1, S, G2, and M. Each of the M/G1, G1 and S phases lasts 2 time points while the G2 phase lasts only one time point, as described in Ramakrishnan *et al.* [15]. Therefore, as shown in Table S3, our method revealed the cell cycles in the YCC data. Since the minimum length of each segment in our approach is set to four (thus, ensuring statistical significance), we could observe coarser segments in comparison to Ramakrishnan *et al.* [15]. Moreover, their algorithm does not account for the statistically significant result as it produced segments of small length (*i.e.*, 3).

Oxidative stress and yeast's cell cycle

In this section, we provide additional elaboration of the results from the different approaches applied on the data capturing the effect of oxidative stress, induced by hydrogen peroxide (HP), on the yeast's

cell cycle. With the filtering step, the number of genes was reduced from 4771 to 1189. The latter were employed to determine the segmentation based on four network properties: degree, betweenness, and closeness, and relative density. Only segments of length at least 4 were considered in order to ensure statistical significance of the Pearson correlation used in network reconstruction. We estimated the thresholds for the Pearson correlation over all considered segment lengths, at significance level $\alpha = 0.05$, by employing empirical permutation test and the randomization procedure from Kruglyak and Tang [33], which allows to consider a dependence structure of adjacent time points.

The characteristics of the resulting segmentations are summarized in Table S4. Based on the work done by Shapira *et al.* [38], with the relative density, which is a global property, we could capture all phases in the system which correspond to the G1, S, G2, G2/M phases of the cell cycle. Although coarser segments are produced by our method than that of Ramakrishnan *et al.* [15], ours is producing statistically significant and robust result due to the minimum length of segments which is set to four. Therefore, this data set further demonstrates that the change of network properties over time caries statistically significant and important biological information.
